# Incomplete Recovery of Pneumococcal CD4 T Cell Immunity after Initiation of Antiretroviral Therapy in HIV-Infected Malawian Adults

**DOI:** 10.1371/journal.pone.0100640

**Published:** 2014-06-24

**Authors:** Enoch Sepako, Sarah J. Glennie, Kondwani C. Jambo, David Mzinza, Oluwadamilola H. Iwajomo, Dominic Banda, Joep J. van Oosterhout, Neil A. Williams, Stephen B. Gordon, Robert S. Heyderman

**Affiliations:** 1 Malawi-Liverpool-Wellcome Trust Clinical Research Programme, University of Malawi College of Medicine, Blantyre, Malawi; 2 Respiratory Infection Group, Liverpool School of Tropical Medicine, Pembroke Place, Liverpool, United Kingdom; 3 Dignitas International, Zomba, Malawi; 4 Division of Clinical Sciences, University of Toronto, Ontario, Canada; 5 Cellular and Molecular Medicine, University of Bristol, Bristol, United Kingdom; South Texas Veterans Health Care System and University Health Science Center San Antonio, United States of America

## Abstract

HIV-infected African adults are at a considerably increased risk of life-threatening invasive pneumococcal disease (IPD) which persists despite antiretroviral therapy (ART). Defects in naturally acquired pneumococcal-specific T-cell immunity have been identified in HIV-infected adults. We have therefore determined the extent and nature of pneumococcal antigen-specific immune recovery following ART. HIV-infected adults were followed up at 3, 6 and 12 months after initiating ART. Nasopharyngeal swabs were cultured to determine carriage rates. Pneumococcal-specific CD4 T-cell immunity was assessed by IFN-γ ELISpot, proliferation assay, CD154 expression and intracellular cytokine assay. *S. pneumoniae* colonization was detected in 27% (13/48) of HIV-infected patients prior to ART. The rates remained elevated after 12 months ART, 41% (16/39) (p = 0.17) and significantly higher than in HIV-uninfected individuals (HIV^neg^ 14%(4/29); p = 0.0147). CD4^+^ T-cell proliferative responses to pneumococcal antigens increased significantly to levels comparable with HIV-negative individuals at 12 months ART (p = 0.0799). However, recovery of the pneumococcal-specific CD154 expression was incomplete (p = 0.0015) as were IFN-γ ELISpot responses (p = 0.0040) and polyfunctional CD4^+^ T-cell responses (TNF-α, IL-2 and IFN-γ expression) (p = 0.0040) to a pneumolysin-deficient mutant strain. Impaired control of pneumococcal colonisation and incomplete restoration of pneumococcal-specific immunity may explain the persistently higher risk of IPD amongst HIV-infected adults on ART. Whether vaccination and prolonged ART can overcome this immunological defect and reduce the high levels of pneumococcal colonisation requires further evaluation.

## Introduction

Invasive pneumococcal disease (IPD), in the form of pneumonia, bacteraemia and meningitis is a leading cause of mortality worldwide [1], [2]. HIV-infected adults and children are 20 to 100 times more likely to suffer invasive pneumococcal disease than age-matched HIV negative persons [3], [4], [5].

Natural protective immunity to *Streptococcus pneumoniae* is thought to rely at least in part on antigen-specific T-cell memory that acts through antibody-dependent and independent pathways that can be rapidly mobilised to mediate microbial clearance at the mucosal surface as well as interrupt multiplication following bloodstream invasion [6], [7], [8], [9]. We have previously shown that mucosal CD4 T-cell immunity to pneumococcal protein antigens is acquired with age and is tightly regulated by antigen-specific CD4^+^CD25^hi^ T regulatory cells [6]. In African high colonisation settings, CD4 T-cell immune memory to these pneumococcal antigens is very commonly detectable in the peripheral blood of adults [10] but appears to be highly susceptible to HIV-mediated immune disruption. Indeed, we have reported that pneumococcal–specific T and B-cell immunity is compromised in HIV-infected Malawian individuals, where there is a high frequency of pneumococcal exposure [11], [12]. We have shown that even in asymptomatic HIV-infected Malawian adults (WHO stage I) pneumococcal-specific interferon–gamma (IFN-γ)-mediated CD4 T-cell effector memory and CD4 T-cell central memory proliferative responses are impaired whilst intrinsic proliferative capacity to PHA remained intact [11]. Although, reconstitution of immunity in general occurs following initiation of antiretroviral therapy (ART) [13], [14], IPD is still 30 times greater in HIV positive persons on ART compared to uninfected individuals [15], [16].

This suggests that following ART, reconstitution of immunity specific to *S. pneumoniae* may be incomplete. Indeed in a cross-sectional study, we have recently shown striking increases in pneumococcal colonization with a broad range of serotypes during the progression of HIV infection in adults [17]. These were associated with dynamic changes in peripheral pneumococcal-specific Th1 IFN-γ immunity which together with these high levels of colonisation did not appear to fully resolve during immune reconstitution with ART.

However, it is important to recognise that the cross-sectional design of our previous study may have led to misleading comparisons between participants with widely differing CD4 nadir's, patterns of pre-ART opportunistic infection and responses to treatment in an African setting. We have therefore investigated the impact of ART on naturally-acquired CD4 T-cell mediated immunity to pneumococcal protein antigens in a prospective longitudinal study of otherwise healthy HIV-infected Malawian adults, using a wider array of functional assays to map out the kinetics and the degree of immune reconstitution, and their relationship to nasopharyngeal carriage of *S. pneumoniae.*

## Materials and Methods

### Ethics Statement

This study complied with institutional practices and guidelines and was approved by the University of Malawi, College of Medicine (Protocol No. P.07/09/801) and Liverpool School of Tropical Medicine (LSTM, Protocol No. 09.71) research ethics committees. Samples (peripheral blood and nasopharyngeal swabs) were obtained following written-informed consent.

### Study design

48 HIV-positive adults initiating ART were recruited from the ART clinic at Queen Elizabeth Central hospital in Blantyre, Malawi and followed up at 3, 6 and 12 months. Eligibility for ART was based on WHO stage 3/4 disease or a CD4 count <250 cells/µl (the cut-off used in Malawi at the time of recruitment).

Participants were excluded from the study if they were: febrile (axillary temperature ≥38.0°C) or unwell; had unexplained weight loss, recurrent respiratory infections, acute Herpes zoster, TB or cryptococcal disease in the past 6 months; were already on ART; had other immunocompromising illness (e.g. diabetes, chronic liver disease, chronic renal impairment, malignancy); known or possible pregnancy; severe anemia (Hb <8 g/dl) or were taking immunosuppressant drugs (e.g. corticosteroids). Samples were also collected from 29 HIV-uninfected healthy individuals (from the same community) and were used to assess the degree of immune reconstitution in the HIV-infected individuals at 12 months ART.

### Plasma HIV-1 Viral load measurement

HIV-1 RNA levels in blood plasma were measured using the Abbot Real-Time HIV-1 assay in a subset of randomly selected participants. Samples were prepared manually and viral load quantified on the Abbot m2000- rt instrument (Abbott Molecular, Germany), according to the manufacturer's instructions (detection level 50 copies/ml).

### Assessment of pneumococcal colonization

Pneumococcal carriage was evaluated using the World Health Organisation standard protocol for detecting nasopharyngeal pneumococcal colonisation [18]. Nasopharyngeal swabs were obtained and immediately placed into a vial of skim milk-tryptone-glucose-glycerol (STGG) transport medium. In the laboratory, the nasopharyngeal swab specimen in STGG media was mixed thoroughly and plated onto a gentamicin (2.5 µg/ml) sheep blood agar (BA) for the isolation of *S. pneumoniae*. The plate was incubated at 37°C, 5% CO_2_ overnight and pneumococci identified by their morphology (alpha-haemolytic colonies) and optochin susceptibility. Susceptibility to optochin was defined as the diameter of inhibition zone. Zones of inhibition greater than 14 mm indicated susceptibility, 7 to 13 mm indicated indeterminate and less than 7 mm as resistant to optochin. Isolates that were susceptible to optochin were considered pneumococci and those that were resistant to optochin were considered to be species other than pneumococcus.

### Cells and Antigens

PBMCs were isolated from blood by 25 minutes centrifugation at 400 g on a density-gradient (Histopaque, Sigma). PBMCs were harvested, washed in hanks balanced salt solution (HBSS, Invitrogen, Paisley, UK) at 400 g for 10 min, and resuspended in complete RPMI (RPMI-1640 with 100 U/ml penicillin, 0.1 mg/ml streptomycin, 4 mM LL-glutamine and 10 mM HEPES buffer). PBMCs were counted using 0.4% (wt/vol) trypan blue (Sigma), reconstituted in complete RPMI at a concentration of 1×10^6^ cells/ml; with a final concentration of 2% (vol/vol) heat-inactivated human AB serum (National Blood Services, Blantyre) [11].

Pneumococcal culture supernatants were prepared from a standard encapsulated type 2 (D39) *S. pneumoniae* strain and an isogenic pneumolysin-deficient mutant (Ply-; was kindly provided by James Paton [19]). The pneumococcal strain was grown on blood agar plates. Colonies showing α-haemolytic pneumococcal type growth and optochin (an antibiotic) sensitivity were then cultured in Todd-Hewitt broth (Oxoid, UK) supplemented with 0.5% yeast extract (THY) in 5% CO_2_ at 37°C to exponential phase, filtered and concentrated. Bradford protein assay was used to measure the concentration of the concentrated pneumococcal culture supernatants (CCS). The concentrated culture supernatants were heat-inactivated at 56°C for 30 minutes to reduce toxic effects of pneumococcal proteins (in the absence of heat inactivation toxicity frequently inhibited proliferative capacity). The absence of Ply in the mutant supernatant was confirmed by discontinuous sodium dodecyl sulphate-polyacrylamide gel electrophoresis and western immunoblotting [10]. The culture supernatants were used at a final concentration of 8 µg/ml.

*Mycobacterium tuberculosis* purified protein derivative (10 µg/ml; PPD RT50) obtained from Statens Serum Institut (Copenhagen, Denmark) and Influenza antigens (0.9 µg/ml) derived from dialyzed inactive trivalent split virion influenza vaccine (2009/2010 vaccine; Sanofi Pasteur MSD Limited, UK) served as reference (respiratory pathogens) controls. Due to limitations in PBMC numbers, not all assays were done on all the subjects at every time point.

### Phenotyping of T cell subsets

200 µl of peripheral blood was stained extracellularly at room temperature in the dark for 10 min with a combination of antibodies specific to each of the three T-cell phenotype groups being assessed (naive/memory T cells, Tregs and Senescent T cells). A combination of CD3 PerCP, CD45RA PE, CD8 APC-Cy7, CD4 APC and CCR7 PE-Cy7 antibodies all from BD Biosciences was used to stain for naive/memory T cells. CD3 PerCP, CD4 pacific blue, CD25 FITC antibodies (BD Biosciences) were used for Tregs. For senescent T cells, peripheral blood was stained with anti-CD3 FITC, anti-CD28 PE, anti-CD4 pacific blue, anti-CD57 APC and anti-CD8 PerCP (BD Biosciences).

After surface staining, red blood cells were lysed with 1× FACS Lysing solution (BD Biosciences) and the cells washed with 1× PBS. The cells surface-stained for Tregs were then permeabilised and fixed using 1× Foxp3 Fixation/Permeabilization working solution as per manufacturer's instructions (ebioscience). After washing with 1× Perm wash (ebioscience), the cells were stained intracellularly with an anti-Foxp3 PE (ebiosciences). The cells were acquired on either a FACSCalibur (BD Biosciences) or a Cyan ADP flow cytometer (Beckman Coulter). Approximately 60 000 events were acquired within the lymphocyte gate.

Data were analyzed with either FlowJo software (TreeStar Inc, Stanford University, FlowJo Africa scheme) or Summit software version 4.3.2 (Beckman Coulter). Lymphocytes were gated based on forward scattering (FSC) and side scattering (SSC) and CD3^+^, CD8^−^, and CD4^+^ cells were considered to be CD4 T cells. These CD4^+^ T cells were analysed for Foxp3^+^ CD25^high^ cells (Tregs), CD45RA and CCR7 expression [naive/memory cells: T_CM_ (CCR7^+^ CD45RA^−^), T_EM_ (CD4^+^ CCR7^−^ CD45RA^−^) and naïve CD4 T cells (CCR7^+^ CD45RA^+^)] and CD57^+^CD28^−^ cells (senescent T cells). Absolute counts of CD4 T lymphocytes were enumerated using BD FACSCount reagent kit (CD4/CD8/CD3) on a BD FACSCount instrument (BD Biosciences).

### IFN- γ enzyme-linked immunosorbent spot (ELISPOT) Assay

Freshly isolated PBMCs were assayed for cells producing IFN-γ, using an ELISPOT assay as previously described [11]. Briefly, Multiscreen HTS 96-Well Filtration plates (Millipore) were coated with 15 ug/ml anti-human IFN-γ mouse monoclonal antibody (1-D1K; Mabtech) in sterile PBS for 4 hours at room temperature. The plates were washed with 1× sterile PBS to remove unbound coating antibody and blocked for 1 hour with 200 µl RPMI supplemented with 10% fetal calf serum (Sigma). PBMCs suspended in complete media were seeded at 0.5×10^6^ cells/well in duplicate wells and incubated with or without antigen for 18 hours at 37°C, 5% CO_2_. Cells were stimulated with D39WT CCS (8 µg/ml), D39Ply- CCS (8 µg/ml). 10 µg/ml PPD (Statens Serum Institute) and 0.9 µg/ml influenza vaccine, split-virion inactivated (2009/2010 vaccine; Sanofi Pasteur MSD Limited UK) were used as reference antigen controls and 5 µg/ml PHA-L (Sigma-Aldrich) as a mitogenic control.

After 18 hours of incubation, the plates were first washed with 1× sterile PBS containing 0.05% TWEEN 20 (Sigma-Aldrich) and then sterile 1× PBS. 1 ug/mL of biotinylated anti–human IFN-γ antibody 7-B6-1 (MABTECH) diluted in 0.5% fetal calf serum plus 1× PBS was added to each well followed by incubated for 2 hours at room temperature. After washing, the cells were incubated with 1 µg/ml of streptavidin-alkaline phosphatase conjugate (Mabtech) for 1 hour at room temperature to detect spot-forming cells (SFCs). Thereafter, the plates were developed using a chromogenic alkaline phosphatase substrate kit (Bio-Rad) as per manufacturer's instruction. Spot forming cell numbers were counted using an ELISPOT plate reader (AutoImmuneDiagnostica, Vers. 4.0). The results were expressed as spot forming units (SFU) per a million PBMCs after subtraction of individual media only background values.

### Proliferation Assay

Lymphocyte proliferation assay was performed as previously described [11]. 12×10^6^ freshly isolated PBMCs were labelled with carboxyfluorescein diacetate 5,6 succinimidyl ester (CFSE dye) (final concentration 1.25 µM, Invitrogen) for 8 minutes at 37°C in the dark. Next, CFSE staining was stopped using 200 µl of fetal bovine serum (Sigma-Aldrich). After washing the cells were resuspended in complete RPMI (RPMI 1640 supplemented with penicillin/streptomycin (concentration, 100 U/mL), L-glutamine (4 mmol/L), HEPES, (10 mmol/L; Sigma-Aldrich) and 2% (vol/vol) of heat-inactivated human AB serum (National blood Services, Blantyre, Malawi).

Labelled cells were cultured at 0.8×10^6^ cells/ml in a 48-well plate with D39WT CCS (8 µg/ml), D39Ply- CCS (8 µg/ml), PPD (10 µg/ml), Influenza (0.9 µg/ml), PHA (5 µg/ml) or media only for 8 days at 37°C, 5% CO_2_. Cells were harvested and stained with anti-CD4-APC and anti-CD8-Percp (BD Biosciences). Finally, the cells were washed with 1× PBS and re-suspended in FACS flow and acquired using the Cell Quest Pro software (version 5.2.1) on a four-colour flow cytometer (BD FACSCalibur). 60 000 events in the lymphocyte gate were acquired. Data were analysed with FlowJo software (TreeStar Inc, Stanford University, FlowJo Africa scheme). Antigen-specific T-cell proliferation was expressed as the percentage of CD4^+^ T cells.

### Detection of CD154 expression as measured by surface mobilization Assay

CD154 expression was measured using a co-culture method as previously described [11], [20]. 1.5–2×10^6^ freshly isolated PBMCs were plated in a 96-well plate in 200 µl RPMI 1640 medium (Sigma-Aldrich) supplemented with 2% heat inactivated AB human serum (National Blood Services, Blantyre, Malawi), 2 mM L-glutamine, 100 U/ml penicillin, 20 mM HEPES buffer and 100 ug/ml streptomycin (Sigma). An anti-CD154-phycoerythrin (PE) antibody was added to every well immediately before stimulation as previously described [20].

The cells were stimulated with D39WT CCS (8 µg/ml), D39Ply- CCS (8 µg/ml), PPD (10 µg/ml), Influenza (0.9 µg/ml), PHA (5 µg/ml) or media only at 37°C, 5% CO_2_ for 16 hours. No costimulatory molecules were added. After stimulation, cells were harvested, washed with PBS and surface-stained with anti-CD3 fluorescein isothiocyanate (FITC), anti- CD4 peridinin chlorophyll protein (PerCP), anti-CD69 allophycocyanin (APC) for 15 min at 4°C in the dark. The cell were then washed with 1 ml 1× sterile PBS and resuspended in FACS flow. The cells were acquired using the Cell Quest Pro software (version 5.2.1) on a four-colour flow cytometer (BD FACSCalibur). Data were analyzed with FlowJo software (TreeStar Inc, Stanford University, FlowJo Africa scheme) by initially gating on lymphocytes followed by CD3 then CD4^+^ cells. CD154 expression was examined on CD69^+^ CD4^+^ T cells.

### Intracellular Cytokine Staining

Intracellular cytokine assay was performed on fresh PBMCs as previously described [21]. 1.5×10^6^ freshly isolated PBMCs were plated in a 96-well plate in 200 µl RPMI 1640 medium supplemented with 2% heat inactivated AB human serum, 2 mM L-glutamine, 100 U/ml penicillin, 20 mM HEPES buffer and 100 µg/ml streptomycin (Sigma).

The cells were stimulated with pneumococcal cell culture supernatants (8 µg/ml) and Purified Protein Derivative (PPD, 10 µg/ml [Statens Serum Institute, Denmark) and influenza vaccine, split-virion inactivated (2009/2010 vaccine; Sanofi Pasteur MSD Limited UK) which were used as reference controls and PMA (10 ng/ml) plus ionomycin (1 µg/ml) (Sigma, St Louis, MO) and incubated at 37°C in the presence of 5% CO_2_.

Brefeldin A (10 µg per ml) (BD Bioscience, UK) was added at 2 h of incubation and the cells were cultured for an additional 16 hr at 37°C, 5% CO_2_. Negative control wells were left unstimulated in all experiments. No costimulatory molecules were added. After stimulation, the cells were harvested, washed and stained with Violet Viability dye (LIVE/DEAD Fixable Dead Cell Stain kit, Invitrogen, UK) for dead cell discrimination as per the manufacturer's instructions. Cells were then stained at 4°C for 15 minutes with surface marker antibodies: CD4 APC-H7and CD8 PerCP all from BD Biosciences.

Next, the cells were washed, permeabilised and fixed using Cytofix/Cytoperm solution (BD Bioscience, UK) as per the manufacturer's instructions. The cells were then stained with anti- IFN-γ APC, anti-TNF-α Alexa Fluor 488 and anti- IL-2 PE (all BD Bioscience, UK) to detect intracellular cytokines. Finally, the cells were washed with 1× Perm Wash (BD Bioscience, UK) and re-suspended in FACS flow. The cells were acquired on a 9 colour Cyan ADP flow cytometer (Beckman Coulter, USA). At least 100 000 lymphocytes were acquired. Cell doublets were excluded using forward light scatter–area vs forward light scatter-height parameters and dead cells discriminated by ViViD. Data were analyzed with FlowJo software (TreeStar Inc, Stanford University, FlowJo Africa scheme) and subsequent analysis was performed using Simplified Presentation of Incredibly Complex Evaluations (SPICE, version 4 (NIAID/NIH,USA); responses were background subtracted and threshold was set at 0.01%). Unstimulated cells were used to set cut-off gates for cytokines.

### Statistical analysis

Statistical analyses and graphical presentation were done using GraphPad Prism 5 (GraphPad). The presence or absence of nasopharyngeal pneumococcal colonization in HIV-infected and uninfected individuals were analyzed using Fishers exact test. Mann-Whitney U test was used for non-paired and Wilcoxon sign ranked test for paired data that was not normally distributed. Differences were considered statistically significant where *P*<0.05.

## Results

### Baseline and follow-up characteristics of study participants

A total of 48 HIV-positive patients initiating ART were enrolled (median age 37 years; [range of 25–55 years]: 21 (44%) males (**Table 1**). All patients started on a generic antiretroviral drug combination of stavudine, lamivudine and nevirapine as well as prophylaxis with cotrimoxazole. During the 12 months post-ART initiation follow-up period, 2 participants died and 6 were lost to follow up. Two patients changed ART regimen due to side-effects. HIV-uninfected persons (29) had similar age and sex distribution to HIV-infected individuals with a systemic median of absolute CD4^+^ T cell count of 602 (range 329–1410) cells/µl.

**Table 1:** Baseline and follow-up characteristics of HIV-infected study participants.

		Time on ART (months)			
	Pre-ART (0)	3	6	12	*P* a
N	48	44	42	40	
Age(Median, range, years)	37(25–55)	NA	NA	NA	
Male[No.%]	21(44)	NA	NA	NA	
CD4 counts					
Median(range), cells/µl	161(3–573)	281(17–737)	249(63–760)	275(73–699)	p<0.001
ARV Treatment [No.]					
stavudine,lamivudine & nevirapine	46				
zidovudine, lamivudine & nevirapine	1*				
stavudine,l amivudine & efavirenz	1*				
Pneumococcal Carriage [No.%]	13/48(27)	14/43(33)	17/42(40)	16/39(41)	0.17

a 0 mth versus 12 mths ART *Changed ART regimen due to side-effects.

**Figure 1:**
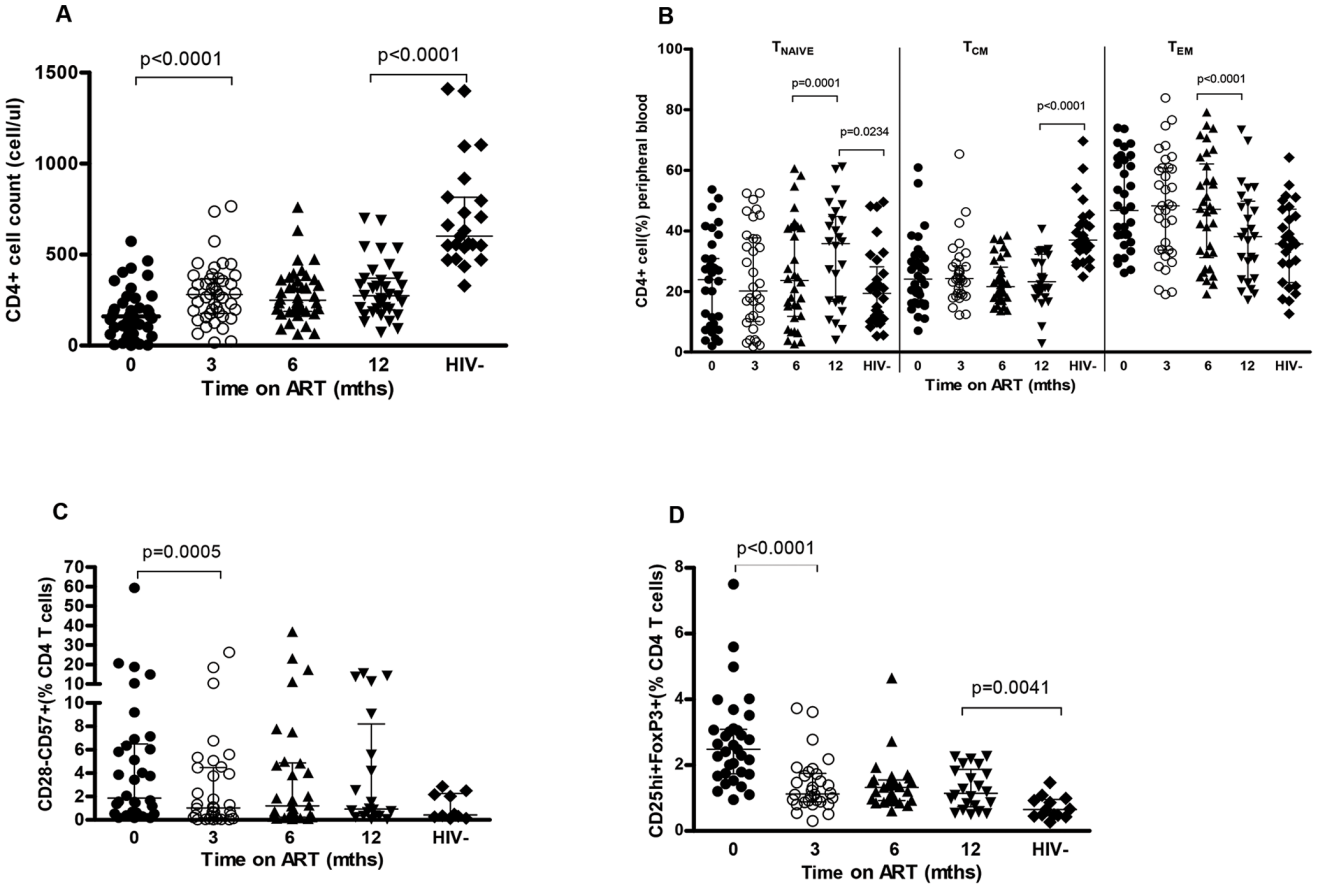
CD4+ T-cell phenotypes in peripheral blood of adults before and after antiretroviral therapy at 3, 6 and 12 months ART and in healthy HIV-uninfected individuals. (**A**) expansion of CD4 T- cell counts (0mth, n = 43; 3mths, n = 43; 6mths, n = 41; 12mths, n = 34; HIV-, 23) (**B**) naive CD4+ T cells (T_N_: CD45RA+CCR7+), central memory CD4+ T cells (T_CM_: CD45RA-CCR7+) and effector memory CD4+ T cells (T_EM_: CD45RA-CCR7-) (0mth, n = 35; 3mths, n = 34; 6mths, n = 34; 12mths, n = 26; HIV-, 29) (**C**) CD28-CD57+ senescent cells (0mth, n = 34; 3mths, n = 34; 6mths, n = 39; 12mths, n = 20; HIV-, 10) (**D**) CD4+CD25^hi^FoxP3+ T regulatory cells (0mth, n = 33; 3mths, n = 30; 6mths, n = 30; 12mths, n = 22; HIV-,13) Black horizontal bars represent median and IQRs. Wilcoxon matched pairs were used to compare T-cell characteristics of HIV-infected persons on ART over time, and the Mann Whitney U test in the HIV-infected and HIV-uninfected comparisons. Representative flow cytometric data demonstrating gating strategy are shown in **Figure S1**.

The median baseline, pre-ART absolute CD4^+^ T-cell count was 161 cells/µl (range 3–573 cells/µl). Baseline CD4^+^ T-cell count categories were: 15 patients had CD4^+^ T-cell counts ≤100 cells/µl; 18 had CD4^+^ cell counts between 101 and 200 cells/µl, 8 had CD4^+^ T-cell counts between 201 and 350 cells/µl and 6 had CD4^+^ T-cell counts >350 cells/µl. The median CD4 T-cell count increased to 275 cells/µl (range: 73–699) at 12 months of ART (p<0.001) (**Table 1**), but remained significantly lower compared to the HIV-negative individuals (**Figure 1A**; median 275 cells/µl vs 602 cells/µl, p<0.0001). The increase in the CD4^+^ T-cell count was most rapid during the first 3 months of therapy (**Figure 1A**; p<0.0001).

Nasopharyngeal carriage of *S. pneumoniae* was detected in 27% (13/48) of study participants at enrolment, increasing but not significantly to 33% (14/43), 40% (17/40) and 41% (16/39) at months 3, 6 and 12 respectively (**Table 1** **, p = 0.17** (pre-ART vs 12 months ART)), but significantly lower than carriage rates in HIV-uninfected individuals (HIV^neg^ 14% (4/29), p = 0.0147[HIV^neg^ vs. HIV^pos12mthsART^]). Viral loads were not routinely available at the time of the study. Plasma HIV-1 RNA was determined for 27 participants (27/43) at 6 months ART. All participants had HIV-1 RNA less than 5000 copies/ml. Eleven had undetectable HIV-1 RNA, nine had 100 - 1000 copies/ml, four had 1000–2000 copies/ml and two had between 2000 and 4000 copies/ml. Further viral load data were not available. However, all participants adhered to a highly effective regimen (nevirapine, stavudine and lamivudine) throughout the study period and none of the patients developed any secondary infection or opportunistic infection to suggest that HIV replication was not being suppressed.

### Changes in CD4 T-cell phenotype during ART

#### Naive and memory T cells

We have previously shown that in asymptomatic HIV-infected individuals (WHO Stage I), the proportion of naive T cells is comparable to healthy HIV-negative persons but memory cells are proportionally decreased [11]. We now show no change in the proportion of naive CD4^+^ T cells (CCR7^+^CD45RA^+^)[21], [22] between 0 and 6 months ART-mediated immune reconstitution but that this subset had further increased by 12 months (**Figure 1B**, p = 0.0001), to levels even higher to those seen in HIV–uninfected individuals (median 35.73% vs 19.36%, p = 0.0234). The proportion of central memory T cells (CCR7^+^CD45RA^−^) remained significantly lower and unchanged for the entire 12 month period compared to HIV-negative individuals (**Figure 1B**, median 23.24% vs. 36.98%, p<0.0001). In contrast, by 12 months ART, the elevated effector memory cell population (CCR7^−^CD45RA^−^) had decreased significantly (**Figure 1B**, p<0.0001[6 months vs. 12 months ART]) to levels similar to those seen in HIV-uninfected individuals (median 38.09% vs. 35.67%, p = 0.5046).

#### Senescent and Tregs CD4^+^ T cells

Chronic immune activation and persistent infections in HIV-1 infected individuals provides an environment that favours accelerated replicative senescence of (CD4^+^CD28^−^CD57^+^) T cells and expansion of CD4^+^CD25^hi+^Foxp3^+^ regulatory T cells (Tregs) [11], [23]. In this African population, we found that the median percentages of CD4^+^CD28^−^CD57^+^ senescent T cells decreased after initiation of ART (month 0: 1.87% [0·17–59·34%], 3 months ART: 1·02% [0·02–26·26%, p = 0.0005] and 12 month ART: 0.92% [0·07–15·45%]) (**Figure 1C**).

The proportion of senescent CD4^+^ T cells was comparable between healthy HIV-uninfected and HIV-infected individuals at 12 months ART (**Figure 1C**, median 0.9200% vs. 0.4200, p = 0.19). Before ART, median percentage of Tregs in HIV-infected patients was 2·45% (0·95–7·5%) of CD4^+^ T cells. The median percentages decreased following initiation of therapy (1.13% [0.30–3.73] at 3 months ART, p<0.0001 and thereafter remained relatively unchanged and significantly higher compared to HIV-negative individuals (**Figure 1D**, median 1,140 vs. 0.6500%, p = 0.0041).

### Functional analysis of CD4 T cells in peripheral blood of adults on ART

#### Regeneration of pneumococcal-specific T-cell ELISpot responses during ART

**Figure 2:**
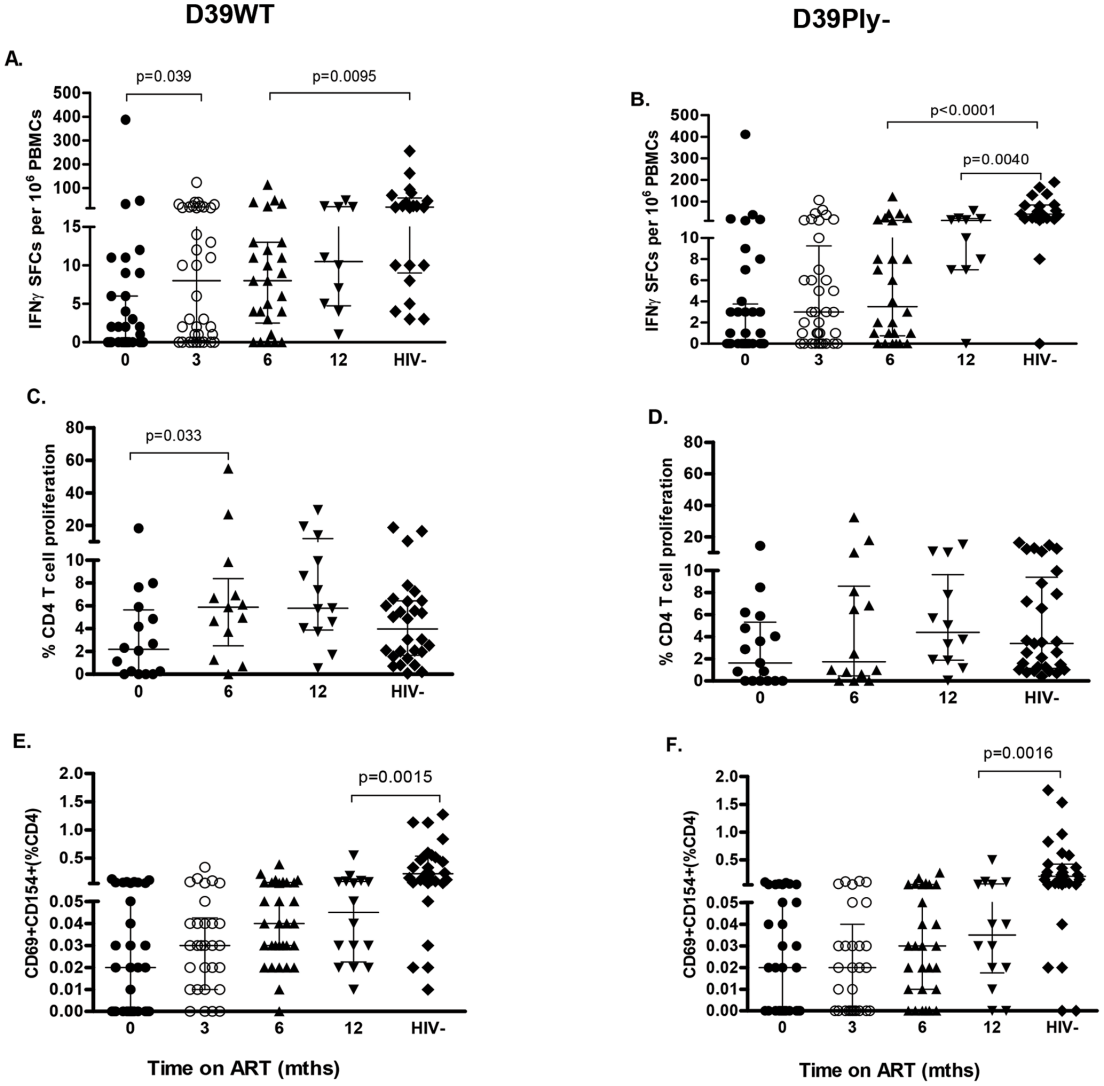
Pneumococcal-specific CD4+ T-cell responses in peripheral blood during ART. Patients were analysed prior to initiation of ART and followed-up at 3, 6 and 12 mths ART for CD4^+^ T-cell responses. Responses from HIV-infected persons at 12 months ART were compared with HIV-negative persons recruited from the same community (**A**) *ex vivo* IFN-γ ELISpot responses to wild-type *Streptococcus pneumoniae* strain concentrated culture supernatant (CCS) (D39WT CCS) (0mth, n = 36; 3mths, n = 36; 6mths, n = 26; 12mths, n = 10; HIV-, 21) (**B**) *ex vivo* IFN-γ ELISpot responses to an isogenic pneumolysin (ply)-deficient mutant (D39Ply- CCS) (0mth, n = 36; 3mths, n = 36; 6mths, n = 26; 12mths, n = 10; HIV-, 21) (**C**) proliferative responses (8 day CFSE dilution assay) to D39WT CCS (0mth, n = 16; 6mths, n = 13; 12mths, n = 13; HIV-, 28) (**D**) proliferative responses to D39Ply- CCS (0mth, n = 17; 6mths, n = 14; 12mths, n = 13; HIV-, 28) (**E**) CD154 expression on activated CD4^+^ T cells in response to D39WT CCS (0mth, n = 33; 3mths, n = 30; 6mths, n = 30; 12mths, n = 16; HIV-, 29) (**F**) CD154 expression on activated CD4^+^ T cells in response to D39Ply- CCS (0mth, n = 30; 3mths, n = 29; 6mths, n = 28; 12mths, n = 14; HIV-, 29). Black horizontal bars represent median and IQR after background responses were substrated from all antigen-specific CD4^+^ T cell responses. Wilcoxon matched pairs were used to compare T-cell characteristics of HIV-infected persons on ART over time, and the Mann Whitney U test in the HIV-infected and HIV-uninfected comparisons. Representative flow cytometric data demonstrating CD4^+^ T-cell proliferative responses and CD154 expression and gating strategy are shown in **Figure S1**.

**Figure 3:**
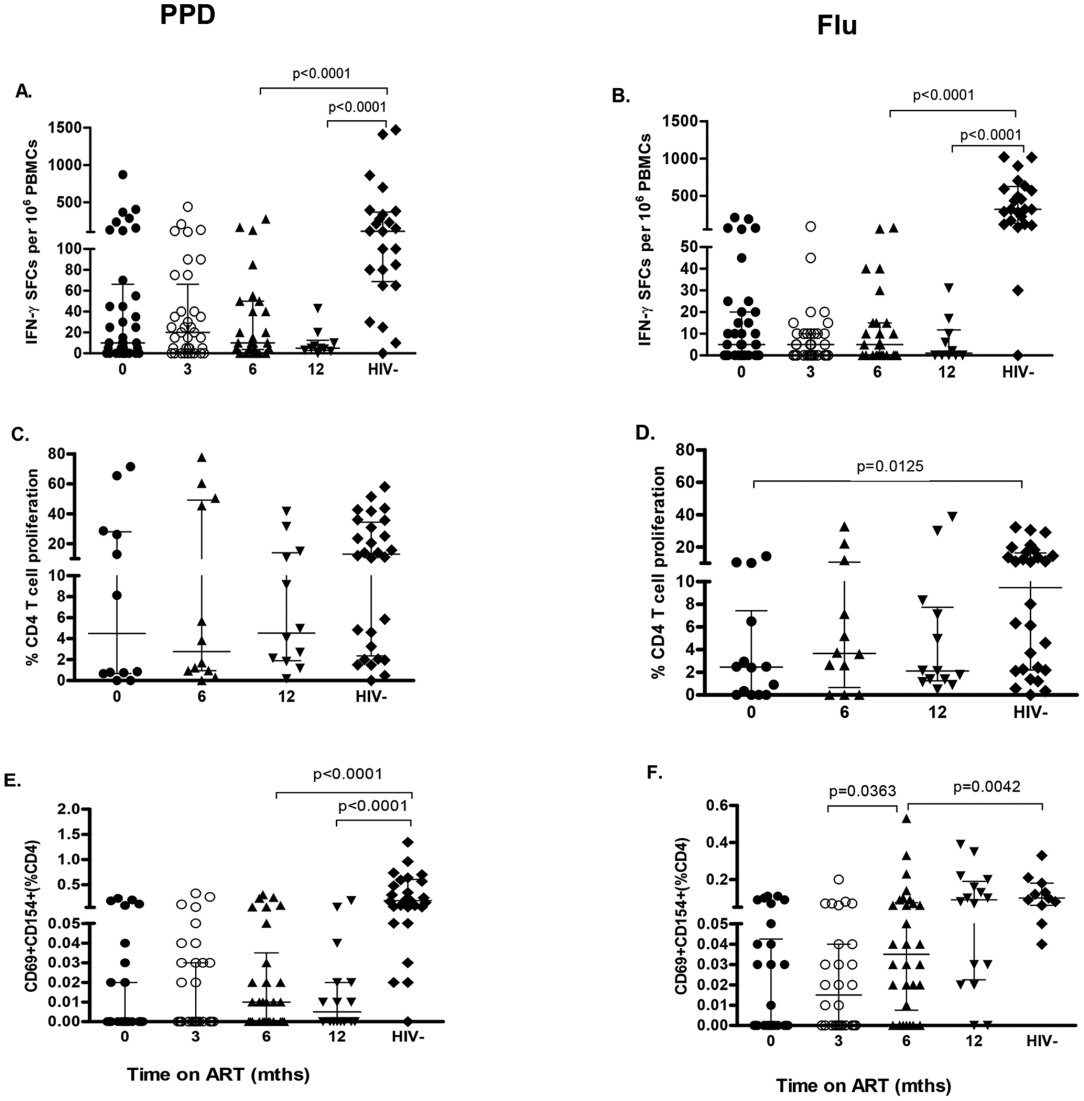
Antigen (PPD and Flu)-specific CD4+ T-cell responses in peripheral blood during ART. Patients were analysed prior to initiation of ART and followed-up at 3, 6 and 12 mths ART for CD4^+^ T-cell responses. Responses from HIV-infected persons at 12 months ART were compared with HIV-negative persons recruited from the same community (**A**) *ex vivo* IFN-γ ELISpot responses to *M.tuberculosis* PPD (0mth, n = 36; 3mths, n = 36; 6mths, n = 26; 12mths, n = 10; HIV-, 24) (**B**) *ex vivo* IFN-γ ELISpot responses to influenza antigens (0mth, n = 36; 3mths, n = 36; 6mths, n = 26; 12mths, n = 10; HIV-, 24) (**C**) proliferative responses (8 day CFSE dilution assay) to *M.tuberculosis* PPD (0mth, n = 12; 6mths, n = 12; 12mths, n = 12; HIV-, 28) (**D**) proliferative responses to influenza antigens (0mth, n = 14; 6mths, n = 12; 12mths, n = 13; HIV-, 28) (**E**) CD154 expression on activated CD4^+^ T cells in response to *M.tuberculosis* PPD (0mth, n = 33; 3mths, n = 30; 6mths, n = 30; 12mths, n = 16; HIV-, 29) (**F**) CD154 expression on activated CD4^+^ T cells in response to influenza antigens (0mth, n = 30; 3mths, n = 28; 6mths, n = 30; 12mths, n = 16; HIV-, 29). Black horizontal bars represent median and IQR after background responses were substrated from all antigen-specific CD4^+^ T-cell responses. Wilcoxon matched pairs were used to compare T cell characteristics of HIV-infected persons on ART over time, and the Mann Whitney U test in the HIV-infected and HIV-uninfected comparisons. Representative flow cytometric data demonstrating CD4^+^ T cell proliferative responses and CD154 expression and gating strategy are shown in **Figure S1**.

We have previously shown that the number of IFN-γ-producing cells in response to pneumococcal antigens is low but detectable in healthy controls and is relatively well preserved in asymptomatic HIV-infected persons [11]. We now show that following initiation of ART, the number of IFN-γ producing cells responding to D39WT CCS (concentrated culture supernatants) increased significantly by 3 months (**Figure 2A**, p = 0.039). By 12 months ART, there was no significant difference between responses detected in HIV-infected individuals and healthy HIV-uninfected subjects (p = 0.1756). Using a CCS from D39Ply-, an isogenic mutant that lacked the immunodominant pneumococcal protein pneumolysin, ELISpot responses were relatively unchanged for the first 6 months of observation (**Figure 2B**, p = 0.13). By 12 months, responses to D39Ply- rose substantially but remained significantly lower compared to HIV-uninfected persons (p = 0.0040). There was no recovery in IFN-γ effector responses to the reference control antigens, PPD and influenza (**Figure 3A and B**; p<0.0001 and p<0.0001 respectively). Changes in any of the T cell responses that we report were not dependent on pre-ART baseline CD4 T-cell count nadir or nasopharyngeal carriage status (data not shown).

#### Pneumococcal-specific T-cell proliferative responses after ART

Following the initiation of ART, the median proportion of proliferating CD4^+^ T cells in response to D39WT CCS increased significantly from 2.197% (IQR, 0.063–5.65) at baseline to 5.88% (2.50–8.38) after 6 months ART (**Figure 2C**, p = 0.033). By 12 months, the proportion of proliferating CD4^+^ T cells was comparable between healthy HIV-uninfected and HIV-infected individuals (**Figure 2C**, p = 0.0799). Immune recovery to D39Ply- CCS was slower but after 12 months ART there was no significant difference between HIV-infected patients and HIV-uninfected controls (**Figure 2D**, p = 0.6161). There were no significant differences between proliferative responses of HIV-infected patients and HIV-uninfected persons in response to PPD and influenza antigens even after 12 months of ART (**Figure 3C & D** respectively).

#### Impaired immune reconstitution of pneumococcal-induced CD154 expression on CD4^+^ T cells during ART

Expression of CD154, a key mediator of T cell-B cell/APC cross-talk, on activated CD4^+^ T cells (CD4^+^CD69^+^ T cells) in response to pneumococcal protein antigens is impaired in HIV infected individuals [11]. Therefore, we investigated whether ART restores this function. By 6 months of ART, the frequency of activated CD4^+^ T cells expressing CD154 in response to D39WT CCS gradually increased compared to baseline (**Figure 2E**, p = 0.05). However, the expression of CD154 in response to D39WT CCS and D39Ply- CCS in HIV-infected persons at 12 months ART were significantly lower compared to HIV-uninfected group (**Figure 2E and F**; p = 0.0015 and p = 0.0016 respectively).

There was little or no recovery of CD154 expression in response to *M. tuberculosis* PPD even after 12 months of ART (**Figures 3E**, p<0.0001), but influenza responses had recovered to levels comparable to the HIV-uninfected group (**Figure 3F**, p = 0.284).

#### Polyfunctional CD4^+^ T cell profile after ART

The presence of T cells capable of simultaneously producing multiple effector cytokines has been associated with productive immune responses to some infections such as leishmaniasis, TB and HIV [24], [25], [26], [27]. However, T cells from HIV-infected persons tend to have impaired ability to simultaneously produce multiple effector cytokines [28] and the frequency of antigen-specific polyfunctional CD4^+^ T cells is reduced during HIV-infection [29]. Therefore, we examined the functionality of pneumococcal-specific CD4^+^ T cells (TNF-α, IL-2 and IFN-γ expression) prior to and after initiation of ART.

**Figure 4:**
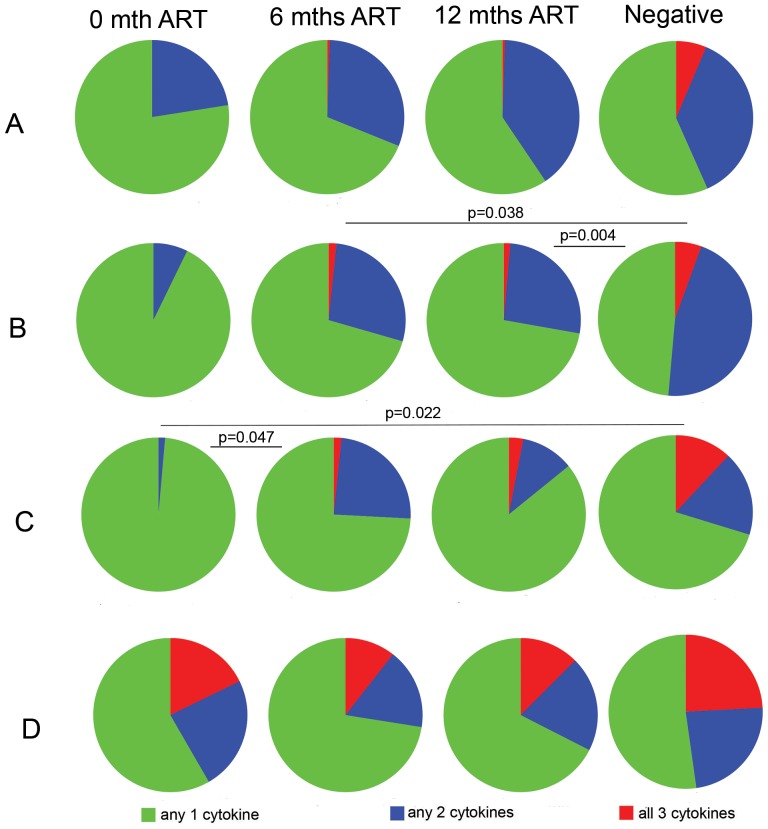
Functionality of CD4+ T cell during ART. Patients were analysed prior to initiation of ART and followed-up at 3, 6 and 12 mths ART for different combinations of IFN-γ, TNF-α and IL-2 using flow cytometry. Polyfunctional CD4^+^ T-cell profiles of HIV-infected persons on ART were compared with HIV-negative persons recruited from the same community. CD4^+^ T-cell responses to (**A**) wild-type *Streptococcus pneumoniae* strain concentrated culture supernatant (CCS) (D39WT CCS) (**B**) an isogenic pneumolysin (ply)-deficient mutant (D39Ply- CCS) (**C**) Influenza vaccine (**D**) *M. tuberculosis* PPD. Collated data (background responses in the negative control substrated and threshold set at 0.01%) from HIV negative (n = 9) and those infected with HIV (n = 18). Pies were analysed according to slice colour using SPICE software and *p* values are indicated. The frequency of CD4^+^ T cells producing one, two or three cytokines specific for D39WT CCS, D39Ply- CCS, influenza vaccine and PPD are shown in **Figure S2**.

At enrolment, neither D39WT nor D39Ply^−^-specific CD4^+^ T cells co-expressed all cytokines (**Figures 4A and 4B** respectively). The functional profile of D39WT-specific CD4^+^ T cells remained unchanged longitudinally after initiation of ART and comparable to the functionality of T cells from HIV-uninfected group. However, D39Ply^−^-specific CD4^+^ T cells from HIV- negative individuals had significantly more T cells producing more than one cytokine simultaneously compared with T cells from HIV-infected patients (**Figure 4B**, p = 0.004). No significant differences in the CD4^+^ profile to PPD and Flu stimulation between 12 months ART and HIV-negative group (**Figure 4C and D** respectively).

## Discussion

HIV infection with or without progression to AIDS, significantly increases the risk of invasive disease due to *S. pneumoniae* [30], [31]. Recently, we showed that pneumococcal-specific T-cell immunity is compromised in asymptomatic HIV-infected Malawian adults, where there is a high frequency of pneumococcal exposure [11]. Here, we extend this previous observation to show the complex pattern of incomplete immune reconstitution in HIV-infected Malawian adults over the course of 12 months of ART. We provide evidence for a defect in pneumococcal-specific CD154 (a key mediator of T-cell/B cell/APC cross talk) expression, T-cell polyfunctionality particularly when the immunomodulatory TLR 4 agonist, pneumolysin [32] was absent from the antigen preparations employed. We also show persistent poor control of pneumococcal nasopharyngeal colonisation.

In the context of CD4 T-cell absolute count recovery and viral suppression, we have found an increase in the proportion of CCR7^+^CD45RA^+^CD4^+^ naive T cells, a decrease in CCR7^−^CD45RA^−^CD4^+^ effector memory T cells and poor recovery of CCR7^+^CD45RA^−^CD4^+^ central memory T cells. These changes were associated with a reduction of CD4^+^ T-cell senescence and a contraction of Tregs after 12 months of ART. Together these findings are highly suggestive of a reduction but not a complete resolution of the generalized immune activation associated with uncontrolled HIV infection.

Despite increases in median CD4 count in subjects established on ART, the rates of colonisation remained high. This is consistent with our recent work which showed HIV-infected individuals who have been on ART for at least 18 months have a significantly higher *S. pneumoniae* carriage rate than HIV-negative persons [17].

Persistent high levels of pneumococcal carriage may be an indication of compromised mucosal immunity (potentially a sign of compromised URT epithelial function or dysregulation of innate and adaptive immunity) which does not reconstitute as fast as blood cellular immunity following initiation of ART. Compromised mucosal immunity may increase the possibility of colonization by a wide range of invasive serotypes and therefore increase vulnerability to IPD. In this study, we did not determine the colonizing pneumococcal serotypes before initiation of ART, whether the same serotypes persisted in patients during the course of the 12 month ART or whether new serotypes were acquired over time. Nonetheless, we have previously investigated serotype composition change during ART in this population [17]. We found that HIV-infected individuals carried a wider range of invasive and non-invasive serotypes compared to HIV-negative persons and that this remained unaltered by ART. In the context of high levels of pneumococcal carriage, we speculate that our study population represents an under-recognised reservoir for genetic exchange and onward pneumococcal transmission.

There were no differences in immune responses between carriers and non-carriers. However, it should be noted that our analyses of carriage were entirely cross-sectional and therefore, do not reflect the true carriage history of the carriers or noncarriers. Additionally, we did not assess the frequency of colonization, duration and density of the carriage events.

In the context of increased CD4 count, reduced antigen load and by extension antigenic stimulation, together with reduced immune activation (contraction of senescent and regulatory CD4 T cells), we show regeneration of pneumococcal-specific IFN-γ ELISpot responses (except D39Ply- responses) and CD4^+^ T-cell proliferative responses. We have previously shown that *ex vivo* IFN-γ ELISpot pneumococcal responses are derived from CD4^+^CD45RO^+^CCR7^−^ effector memory cells [10]. Therefore, a recovery of IFN-γ responses reflects at least partial regeneration of the pneumococcal-specific T_EM_ function.

In healthy African populations, high levels of pneumococcal carriage stimulate cellular immunity at the mucosal level which can be detected in the blood [10]. Indeed in a cross-sectional study, we have recently shown that increased colonization coincided with dynamic changes in peripheral pneumococcal-specific Th1 IFN-γ immunity in HIV-symptomatic individuals [17]. Whether the modest reconstitution of pneumococcal-specific IFN-γ ELISpot and proliferative T-cell responses seen in our study population can be attributed at least in part to increases in pneumococcal colonisation remains to be established.

We have demonstrated impaired upregulation of CD154 (a key mediator of T-cell/B cell/APC cross talk). Given the changes in pneumococcal-specific IFN-γ ELISpot and proliferative T-cell responses following initiation of ART, the CD154 data somewhat implicate defects in T-B signalling. Indeed, CD154 deficient T cells homing to B follicles will fail to facilitate B- cell production of pneumococcal-specific antibodies [33]. We also demonstrated modest reconstitution of influenza-specific responses during ART possibly due to less frequent and intense antigen exposure, consistent with a previous study performed in the same setting [34].

Given the selective regeneration of pneumococcal-specific T cells even after 12 months of ART, such individuals may need additional protection principally through vaccination. Because of the suboptimal activity of the 23-valent pneumococcal polysaccharide vaccine in HIV-infected African adults [35], routine PPV23 vaccination of HIV-infected adults is not recommended for use in resource poor countries [36]. A recent study in Malawi, provided evidence that the 7-valent pneumococcal conjugate vaccine (PCV) protected HIV-infected adults from recurrent pneumococcal infection caused by vaccine serotypes, thus supporting a role for conjugate vaccines among HIV-infected adults [37].

Several studies have shown that following ART initiation, there is often poor restoration of antigen-specific immune responses in individuals who are severely immunodeficient prior to treatment [38], [39], [40], [41] However, although a low CD4 pre-treatment nadir was common (68% of the study participants presented for the first time with CD4 counts <200 µl) there was no clear association in our cohort.

T cells that produce multiple factors simultaneously (e.g. TNF-α, IL-2 and IFN-γ) are termed polyfunctional T cells and have been shown to provide protection against diseases in murine models of Leishmaniasis and TB [24], [42], control of hepatitis C and HIV-1 infection [28] and possibly vaccine-induced immunity [43], [44]. In contrast to the almost full recovery of pneumococcal (D39)-specific polyfunctional T cell responses, D39Ply- responses remained defective. These data were consistent with the IFN-γ ELISpot data which showed little recovery of D39Ply- IFN-γ ELISpot responses at 12 months ART. These data also fits well with what we have shown before that immune memory to the pneumococcus is differentially influenced by antigen [10]. Therefore, the role of pneumococcal protein antigens in immune recovery during ART requires further investigation.

When this study was started, most patients were starting ART with CD4 T-cell counts less than 200 cells/µl. However, recently more patients are being started on therapy at high CD4 T-cell counts and therefore whether the observations we report here also hold true for these patients will need to be confirmed.

In conclusion, we have demonstrated poor control of pneumococcal colonization in HIV-infected persons which persists despite ART. Despite some restoration of pneumococcal-specific CD4 T-cell proliferation, we found impaired pneumococcal-specific CD154 expression as well as incomplete recovery of D39Ply- IFN-γ ELISpot responses and CD4 T-cell polyfunctionality. These results together with observations we have made previously [11], [17] provide the rationale for further longitudinal studies of pneumococcal carriage and immunity to better define the mechanisms of immune control. In particular, to explore the relationship between pneumococcal-specific cellular immunity detected in the blood, and pathogen clearance and immune surveillance at the mucosal surface in both immunocompetent and immunocompromised hosts.

## Supporting Information

Figure S1 **Representative flow cytometric data (CD4+ T cells) and gating strategy** (**A**) Plot showing naive and memory CD4 T-cell subsets gated on characteristic expression patterns of CD45RA and CCR7 (Upper left quadrant T_CM_ - central memory CD4^+^ T cells, lower left quadrant T_EM_ – effector memory CD4^+^ T cells and upper right quadrant T_N_– naive CD4^+^ T cells) in peripheral blood (**B**) Plot showing phenotypic analysis of CD25 and FoxP3 expression in peripheral blood (**c**) Plots showing expression of CD69 and CD154 in media only, pneumococcal antigens, *M. tuberculosis* PPD and PHA (**D**) CD4^+^ T-cell proliferative responses in media, pneumococcal antigens, *M. tuberculosis* PPD and PHA (**e**) Plots showing cytokine expression following stimulation with pneumococcal antigens and *M. tuberculosis* PPD.
(TIF)

Figure S2 **Functionality of CD4+ T cells after ART.** Patients were analyses prior to initiation of ART and followed-up at 3, 6 and 12 mths ART for different combinations of IFN-γ, TNF-α and IL-2 using flow cytometry and SPICE software (version 4). Charts show the frequency of CD4^+^ T cells producing one, two or three cytokines specific for (**A**) wild-type *Streptococcus pneumoniae* strain concentrated culture supernatant (CCS) (D39WT CCS) (**B**) an isogenic pneumolysin (ply)-deficient mutant (D39Ply- CCS) (**C**) Influenza antigens (**D**) *M. tuberculosis* PPD.
(TIF)
