# Supplementary figures and images for: Incomplete Recovery of Pneumococcal CD4 T Cell Immunity after Initiation of Antiretroviral Therapy in HIV-Infected Malawian Adults
Source: PLoS One. 2014 Jun 24;9(6):e100640. doi: 10.1371/journal.pone.0100640 (PMC4069109; doi:10.1371/journal.pone.0100640)

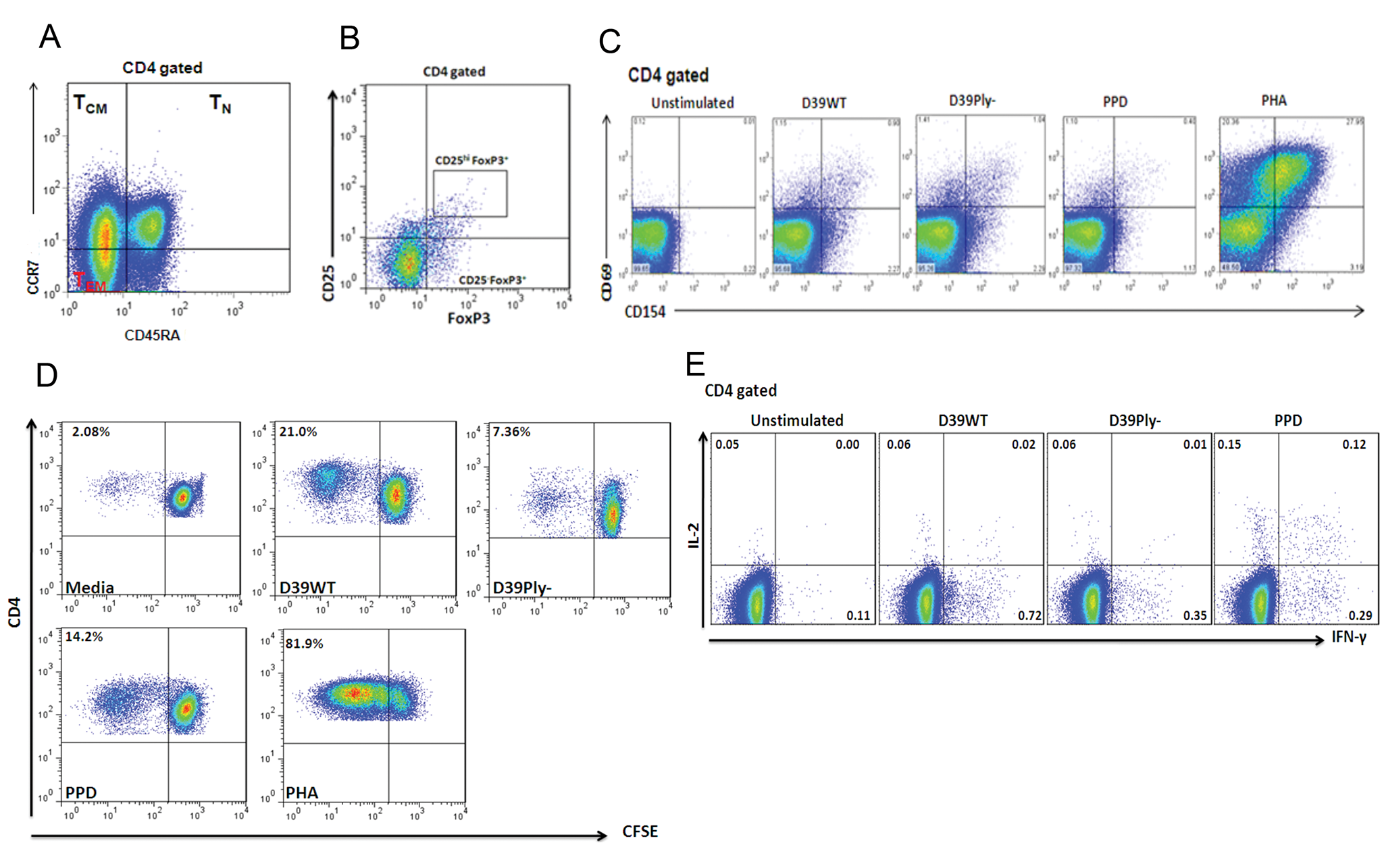

Supplement: Figure S1 — Representative flow cytometric data (CD4+ T cells) and gating strategy (A) Plot showing naive and memory CD4 T-cell subsets gated on characteristic expression patterns of CD45RA and CCR7 (Upper left quadrant TCM - central memory CD4+ T cells, lower left quadrant TEM – effector memory CD4+ T cells and upper right quadrant TN– naive CD4+ T cells) in peripheral blood (B) Plot showing phenotypic analysis of CD25 and FoxP3 expression in peripheral blood (c) Plots showing expression of CD69 and CD154 in media only, pneumococcal antigens, M. tuberculosis PPD and PHA (D) CD4+ T-cell proliferative responses in media, pneumococcal antigens, M. tuberculosis PPD and PHA (e) Plots showing cytokine expression following stimulation with pneumococcal antigens and M. tuberculosis PPD. (TIF) [file pone.0100640.s001.tif]

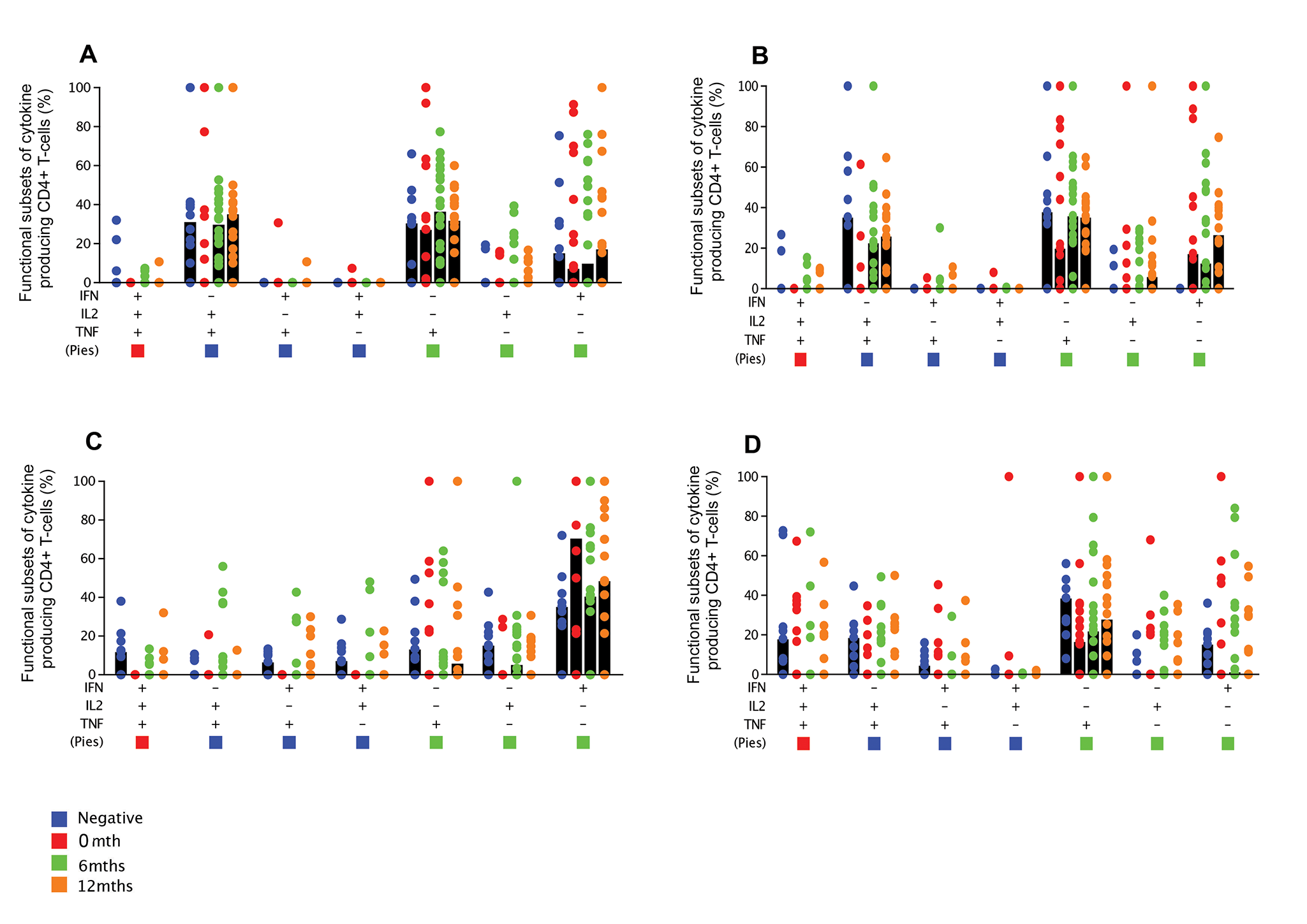

Supplement: Figure S2 — Functionality of CD4+ T cells after ART. Patients were analyses prior to initiation of ART and followed-up at 3, 6 and 12 mths ART for different combinations of IFN-γ, TNF-α and IL-2 using flow cytometry and SPICE software (version 4). Charts show the frequency of CD4+ T cells producing one, two or three cytokines specific for (A) wild-type Streptococcus pneumoniae strain concentrated culture supernatant (CCS) (D39WT CCS) (B) an isogenic pneumolysin (ply)-deficient mutant (D39Ply- CCS) (C) Influenza antigens (D) M. tuberculosis PPD. (TIF) [file pone.0100640.s002.tif]
